# The Efficacy and Immunomodulatory Effects of Ulinastatin and Thymosin *α*1 for Sepsis: A Systematic Review and Meta-Analysis

**DOI:** 10.1155/2016/9508493

**Published:** 2016-05-31

**Authors:** Feng Yun Wang, Bin Fang, Xin Hua Qiang, Tie Ou Yu, Jia Rong Zhong, Jun Cao, Li Xin Zhou

**Affiliations:** First People's Hospital of Foshan, Critical Care Department, Foshan 528000, China

## Abstract

*Objective*. To systematically review the efficacy and potential immunomodulatory effect of ulinastatin combined with thymosin *α*1 (UTI) for sepsis.* Design*. A systematic review and meta-analysis of randomized controlled trials (RCTs).* Data Sources*. The following databases: PubMed, Embase, and Cochrane Central were searched to identify related clinical trials. The search terms were “ulinastatin”, “thymosin”, and “sepsis”.* Results*. Six RCTs, 944 septic patients in total, were included in this meta-analysis. The result shows UTI increased the 28-day survival rate of septic patients, odds ratio (OR) = 2.01, 95% CI [1.53, 2.64]. After the treatment with UTI, the APACHE II score (four studies) dropped 4.72 further, mean = −4.72, 95% CI [−6.54, −2.91] (*p* < 0.00001). The mean time of ICU stay (four studies) in UTI group decreased 3.03 days further, mean = −3.03 [−6.99, 0.95] (*p* = 0.14), and mechanical ventilation time (four studies) decreased 2.05 days, mean = −1.81 [−2.96, −0.66] (*p* = 0.002). With the treatment of UTI, CD4+T cells raised 5.13%, mean = 5.13, 95% CI [2.75, 7.50] (*p* < 0.0001); there was no significant change in CD8+T cells, mean = −0.74 [−2.93, 1.45] (*p* = 0.51).* Conclusion*. According to this meta-analysis, with the treatment of UTI, the short-term survival rate of septic patients was increased and the illness severity was alleviated. ICU stay and mechanical ventilation time were effectively shortened. The beneficial effect of UTI might be due to the potential immunomodulatory effects of these two drugs.

## 1. Introduction

Systemic illness caused by microbial invasion of normally sterile parts of the body is referred to as “sepsis,” which is an increasingly common cause of morbidity and mortality, particularly in the elderly, immunocompromised, and critically ill patients [1, 2]. Sepsis develops in 750,000 people annually, and more than 210,000 of them die in US [3]. A cohort study shows that mortality ranged from 28.3% to 41.1% on different continents [4].

After numerous unsuccessful trials of anti-inflammatory agents in patients with sepsis through the years, investigators doubted whether the mortality could be decreased. For although bacteria play a leading role in sepsis, the direct cause of death for patients with sepsis is not the bacteria themselves but rather the persistent paralysis of the immune system and the excessively magnified systemic inflammatory response.

Thus, the clinical researchers in Asia turned to immune-modulating reagents like ulinastatin and thymosin *α*1, calculating that these 2 reagents might prevent the inflammation spreading too fiercely while simultaneously enhancing the body's immunity to avoid immune paralysis. Ulinastatin is a multivalent, Kunitz type serine protease inhibitor that is found in human urine and blood [5]. It has an inhibitory effect not only on trypsin and a-chymotrypsin, but also on leukocyte elastase hyaluronidase activity. With its potential anti-inflammation effect, ulinastatin was used to treat a wide-spectrum of diseases [6–11]. Thymosin *α*1 is an acidic peptide purified from calf thymus tissue and has an acetylated amino-terminus, which is a potent inducer of T cells [12]. Notably, a multicenter RCT shows T*α*1 therapy could lower short-term, all-cause mortality and promote the percentage of HLA-DR antigen expressed on lymphocytes in patients with severe sepsis [13].

The combination administration of ulinastatin and thymosin *α*1 (UTI) may regulate inflammation bidirectionally, which may simultaneously prevent immunologic paralysis and prevent excessive inflammation. With such a hypothesis, many clinical trials were carried out in Asia to observe the efficacy of UTI for sepsis. The objective of this paper is to quantify the efficacy and immune-modulatory effect of UTI for sepsis by systematic reviewing and meta-analysis. The endpoints in this paper are 28-day survival rate, APACHE II difference, ICU stay, mechanical ventilation time, CD4+T cell percentage, and CD8+T cell percentage.

## 2. Methods

### 2.1. Data Sources

PubMed (up to December 8th 2014), Embase (up to December 2014), and Cochrane Central (Issue 9 of 12 September 2014) were searched to find relevant clinical trials with a prespecified search strategy, which was revised appropriately through the databases. Trials other than a randomized controlled trial (RCT) were eliminated from consideration. Search terms included “ulinastatin”, “thymosin”, “sepsis”, ”mortality”, “survival rate”, and “efficacy”. These were combined by patients, intervention, control, and outcomes (PICOs) principle to formulate a search strategy. The search strategy is as follows: “(((((((sepsis[Title/Abstract]) OR septic shock[Title/Abstract]) OR infection[Title/Abstract]) OR infectious disease[Title/Abstract])) AND (((ulinastatin) OR trypsin inhibitor) OR thymosin)) AND ((((((RCT[Title/Abstract]) OR randomized controlled trial[Title/Abstract]) OR randomized[Title/Abstract]) OR controlled[Title/Abstract]) OR placebo[Title/Abstract]) OR sham[Title/Abstract])) AND ((((mortality[Title/Abstract]) OR death rate[Title/Abstract]) OR survival rate[Title/Abstract]) OR efficacy[Title/Abstract])” in PubMed and it is adjusted accordingly in other databases. English and Chinese scientific literature databases were thoroughly accessed in the searching process. Emails were sent to corresponding authors to inquire about relevant studies and to pharmaceutical companies to gather information about any ongoing RCTs related to ulinastatin or thymosin *α*1.

### 2.2. Study Selection

Two authors (Feng Yun Wang and Li Xin Zhou) independently searched and examined the relevant literatures and scanned the title and abstract of retrieved articles to evaluate them for further assessment. Full articles were obtained when the information given in the titles and abstracts implied that the study included an RCT research design intended to investigate the efficacy of ulinastatin and thymosin *α*1 for sepsis. When disagreements occurred, they were discussed thoroughly to reach a consensus. The criteria for inclusion were as follows: (1) any RCT concerns ulinastatin combined with thymosin *α*1 for sepsis; (2) the patients enrolled were of any age and any gender and had a confirmed sepsis; (3) ulinastatin intravenously infused with any dosage and thymosin *α*1 subcutaneously injected with any dosage.

### 2.3. Qualitative Assessment

The quality of the included literatures was assessed by the Cochrane Collaboration tool for assessing risk of bias, which contains the following 5 aspects: sequence generation, allocation concealment, blinding, incomplete outcome data, and selective outcome reporting. The assessment of risk of bias was presented by using a “risk of bias summary figure,” which presents all of the judgments in a cross-tabulation of study by entry. This display of internal validity indicates the weight the writer may give to the results of each study.

### 2.4. Data Extraction

Two review authors independently extracted data and the process included the following detailed information: (1) the name of authors, year of publication; (2) intervention measures, including dosage, route, and timing; (3) number of intention-to-treat (ITT) patients in both groups (trial and control); (4) endpoints were 28-day survival rate, APACHE II score difference, ICU stay, mechanical ventilation time, CD4+T cell percentage, and CD8+T cell percentage. The data of dichotomous variables such as the number of 28-day survival patients were directly extracted from the included literatures. The meta-analysis data of continuous variables such as APACHE II score difference, CD4+T cell percentage, and CD8+T cell percentage were calculated according to Cochrane Handbook's methodology. For example, *X* = |*X*_2_ − *X*_1_|, where *X* represents the mean applied in this meta-analysis; *X*_2_ represents endpoint mean; and *X*_1_ is the baseline mean of specific variable in included studies. In *S* ^2^ = *S*_1_^2^ + *S*_2_^2^ − 2*∗R∗S*_1_ *∗S*_2_, *S* represents the standard deviation (SD) applied in this meta-analysis; *S*_2_ represents the endpoint SD; and *S*_1_ represents the baseline SD of specific variable presented in the included studies. *R* is a constant, usually possessing a value of 0.4 or 0.5 in meta-analysis, as found in the Cochrane Handbook. In this meta-analysis, the value of *R* is 0.5.

### 2.5. Data Analyses and Statistical Methods

Data analyses of this review were performed by Review Manager 5.3 (Version: 5.3.5, Cochrane Collaboration, UK). Clinical heterogeneity was assessed in the population, methodology, and intervention and outcome measures of each study to evaluate whether pooling of results was feasible. Heterogeneity assessment was performed using the *χ* ^2^ test, where a *p* value less than 0.1 was considered as the significance set. Values of *I* ^2^ less than 25% were deemed to have low heterogeneity, and a fixed-effect model for meta-analysis was then used. Value of *I* ^2^ more than 25% was deemed to have considerable heterogeneity, and a random effects model was then utilized [20]. All statistical tests were two sided and a *p* value less than 0.05 was considered statistically significant.

## 3. Results

### 3.1. Study Selection Process

**Figure 1:**
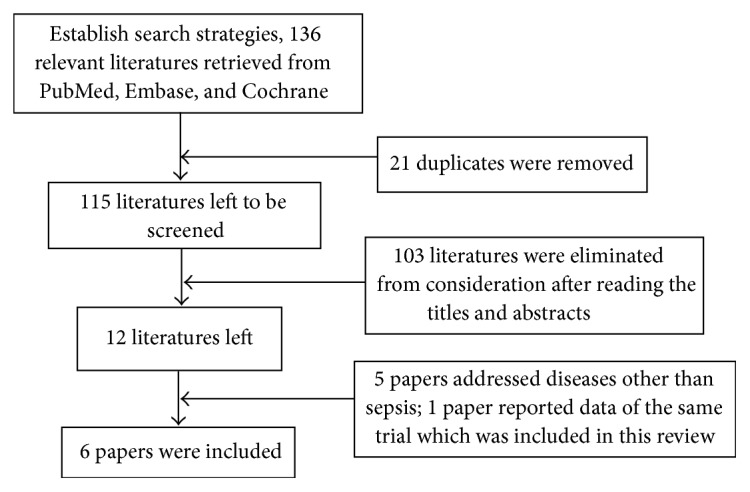
Study flow diagram for relevant randomized controlled trials.

The flow diagram in Figure 1 shows the complete scanning and selection process. In total, 136 articles were retrieved from electronic databases. 115 articles were retained to read the title and abstract, after deleting duplicates. The full text of 12 articles was thoroughly read to further review after scanning. Finally, 6 of the 12 articles met the inclusion criteria [14–19].

### 3.2. The Characteristics of the Included Literatures

**Figure 2:**
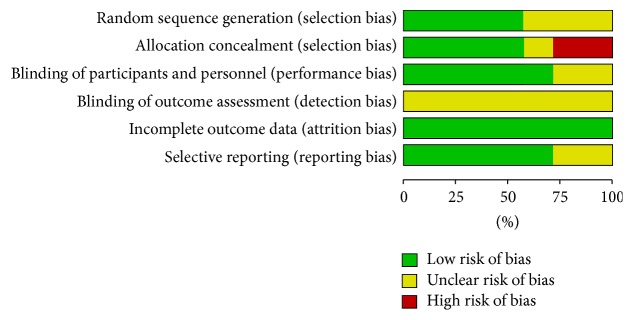
Risk of bias graph, review authors' judgments about each risk of bias item presented as percentages.

The six studies included all were RCTs, three of the six utilized opaque envelopes for randomization [14, 17, 18]. The other three did not specify their means of randomization. Four of the six papers stated clearly that placebos were used in the trials [14–17], whereas the other two did not indicate if placebos were used or just blank control in the trials [18, 19]. Of particular note, neither personnel nor evaluator blindness were mentioned in any of the included studies. No attrition bias was detected in any of the six studies. Information such as the type of study, route of medication, duration of treatment, and sample size was listed in Table 1. Bias risks were specified in Figure 2.

### 3.3. Meta-Analyses Results

#### 3.3.1. 28-Day Survival Rate

**Figure 3:**
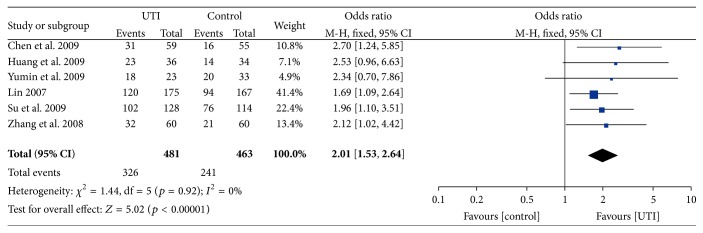
Meta-analysis of 28-day survival rate compares UTI with conventional therapy for sepsis. The vertical line suggests no difference between UTI and conventional therapy. The size of each square represents the proportion of information given by each trial.

The six included RCTs enrolled a total of 944 patients. Since no obvious heterogeneity was detected between the studies, a fix-model was adopted, and the pooling result showed that UTI could increase the 28-day survival rate, OR = 1.91, 95% CI [1.47, 2.47] (*p* < 0.00001) Figure 3.

#### 3.3.2. 28-Day APACHE II Score Difference

**Figure 4:**
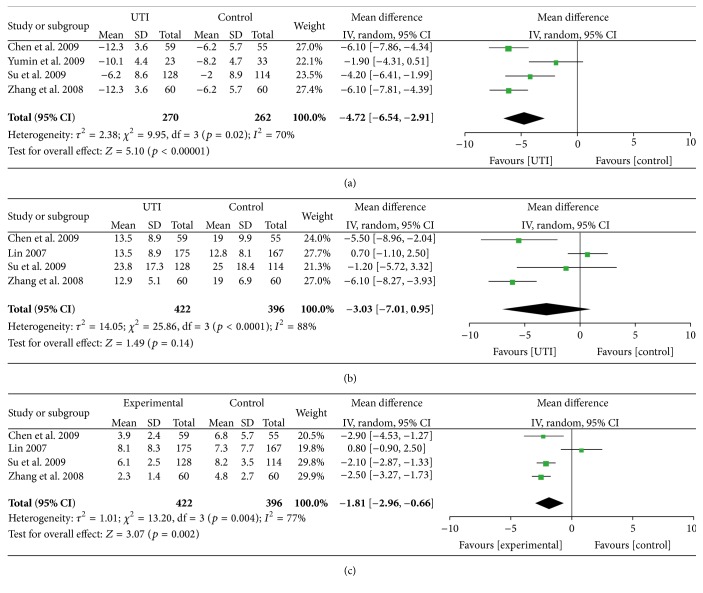
Meta-analysis of APACHE II score difference, ICU stay, and mechanical ventilation time compares UTI with conventional therapy for sepsis: (a) APACHE II score difference; (b) ICU stay; (c) mechanical ventilation time. The vertical line suggests no difference between UTI and conventional therapy. The size of each square represents the proportion of information given by each trial.

APACHE II score difference values were successfully extracted from four RCTs, with a total of 526 patients enrolled, and considerable heterogeneity was detected. Thus, a random-model was adopted when synthesizing the result, which showed that 28 days after treatment, when compared to control group, the APACHE II score decreased 4.72 comparatively, mean = −4.72, 95% CI [−6.54, −2.91] (*p* < 0.00001) (Figure 4(a)).

#### 3.3.3. ICU Stay

 Four RCTs, with 812 patients enrolled, reported ICU stay. The heterogeneity of these was high (*I* ^2^ = 88%), so a random-model was applied. The pooling result showed UTI can shorten ICU stay by 3.02 days, mean = −3.03 [−7.01, 0.95] (*p* = 0.14) (Figure 4(b)).

#### 3.3.4. Mechanical Ventilation Time

Four RCTs with 812 patients enrolled reported mechanical ventilation time. Since these four had considerable heterogeneity between them, a random-model was used. The pooling result revealed that UTI can reduce mechanical ventilation time by 2.05 days, mean = −2.05 [−3.17, −0.93] (*p* = 0.002) (Figure 4(c)).

#### 3.3.5. Immunomodulatory Effects

**Figure 5:**
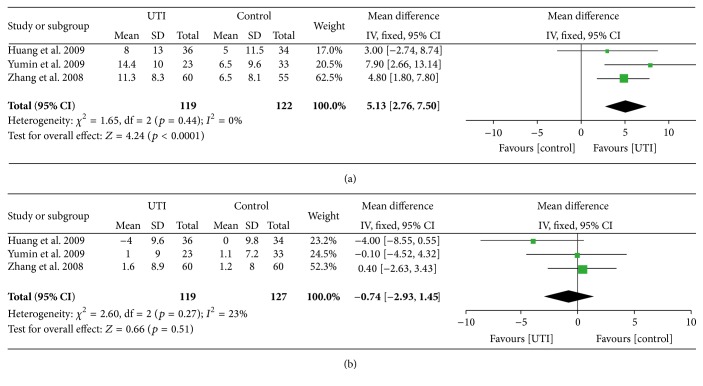
Meta-analysis of CD4+T cell percentage (a) and CD8+T cell percentage (b) compares UTI with conventional therapy. Vertical line suggests that no difference exists between UTI and conventional therapy. The size of each square represents the proportion of information given by each trial.

Three RCTs reported data about CD4+T, CD8+T, and these three included a total of 246 patients. With the treatment of UTI, CD4+T cells percentage was raised 5.13% comparatively, mean = 5.13, 95% CI [2.76, 7.50] (*p* < 0.0001) (Figure 5(a)). CD8+T cells percentage had no significant change when compared to control group after the treatment of UTI, mean = −0.74 [−2.93, 1.45] (*p* = 0.50) (Figure 5(b)).

## 4. Discussion

To our knowledge, this is the first systemic review and meta-analysis about ulinastatin combined with thymosin *α*1 for sepsis. Four out of the six RCTs were in English [14–17], while the other two were published in Chinese scientific journals [18, 19]. Nevertheless, the latter's abstracts were also available in English and discoverable at Medline. The six included RCTs enrolled septic patients and had a similar inclusion criteria [21, 22], all timely following Surviving Sepsis Campaign guidelines. With regard to the administration route, ulinastatin was intravenously infused and thymosin *α*1 was subcutaneously injected in all the six RCTs; therefore, it is appropriate to say the UTI treatment had a similar pharmacokinetics in the included trials. Five RCTs treated patients with a duration of 6 days [14–18] and one for 10 days [19]. Five RCTs had a reduced dosage in the second half treatment course [14–17, 19], while the other trial had a constant dosage throughout the trial [18]. Based on the above analysis, it might be appropriate to say though there were slight differences amongst the trials, no significant heterogeneity existed in the designing of the included trials.

The result of our meta-analysis suggests that UTI treatment could significantly increase the short-term survival rate of the sepsis patients, OR = 2.01, 95% CI [1.53, 2.64] (*p* < 0.00001). Notwithstanding the leading role played by antimicrobial reagents in treating sepsis, with their anti-inflammatory and potential immunomodulatory effects, ulinastatin and thymosin *α*1 treatment, according to the data, enhanced the survival opportunities of sepsis patients significantly. Nonetheless, since only 944 patients were enrolled in the meta-analysis, the data interpretation should be carried out prudently.

With systematic treatment, the APACHE II score dropped dramatically in both UTI group and control group; however, with the treatment of UTI, the APACHE II score further decreased 4.72 when compared with control group, mean = −4.72, 95% CI [−6.54, −2.91] (*p* < 0.00001). On the strength of the above data, we might conclude that UTI treatment could ameliorate the severity of sepsis, which is consistent with its efficacy in promoting 28-day survival rate. Furthermore, the pooling results indicated that UTI treatment could shorten ICU stay by 3.03 days, mean = −3.03 [−7.01, 0.95] (*p* = 0.14); however, the result did not reach statistical significance; and it could shorten mechanical ventilation (MV) time by 1.81 days, mean = −1.81 [−2.96, −0.66] (*p* = 0.002). The reduction of both ICU stay and MV time could effectively lower the medical cost as well as save plenty of medical resources.

Furthermore, a protective effect of ulinastatin was also observed in relation to other diseases. In major hepatectomy, ulinastatin could protect liver function and improve clinical outcomes, possibly via the inhibition of inflammation and oxidation at an earlier stage [6]. Prophylactic short-term administration of ulinastatin was found to decrease the incidence of pancreatitis and hyperenzymemia after ERCP [7–10]. It was also reported that ulinastatin prevents new organ dysfunction and reduces mortality in subjects with severe pancreatitis [11]. Clinical trials demonstrate that, for other diseases which necessitate heart surgeries, especially cardiopulmonary bypass, short-term usage of ulinastatin could ameliorate myocardial injury and shorten the time of tracheal intubation and ventilation [23–25]. In patients with severe traumatic brain injury, ulinastatin treatment could effectively improve cerebral oxygen metabolism and reduce CRP level [26].

With regard to thymosin *α*1, it was demonstrated that prophylactic usage of it could lower the incidence of infection in patients with nephrotic syndrome [27]. In patients with severe acute pancreatitis, administration of thymosin *α*1 could improve cell-mediated immunity and reduce the occurrence of infection [28]. With its potential immune-modulatory effect, thymosin *α*1 was used to treat hepatitis B patients in some clinical trials and its efficacy was proved by pathological observation [29]. The results showed that treatment with thymosin *α*1 increased cytokine production, especially IFN-*γ* and, at a higher-dose, thymosin *α*1 exhibited better efficacy against HBV, compared with other treatments studied. Evidence shows that thymosin *α*1 may improve resistance to infection and induce immune tolerance without GVHD in hematopoietic stem cell transplantation patients [30].

In this meta-analyses, three of the included RCTs reported data about CD4+T cells and CD8+T cells. The pooling results showed that the percentage of CD4+T cells gradually recovered after the treatment of UTI, which was consistent with former studies that conclude that UTI could improve cell-mediated immunity. The recovering percentage of CD4+T cells is a robust symbol of the ability to restore immune function in clinical situations. Since only three trials were enrolled in the analysis, UTI's potency of regulating immunity still lacks high-level evidence. It is worth mentioning that though UTI was regularly administered for severe sepsis in Asian countries, no severe side effects have been reported so far and no drug-related events were reported in the included RCTs either.

Cost-effectiveness not only concerns doctors who are desperately trying to save septic shock patients, it also concerns the families of the patients. Unfortunately, no cost-effectiveness data were revealed by any author in the included studies. To our knowledge, ulinastatin costing approximately $12/50000 U, 600000 U/day for 7 days will increase medical bills by nearly $1000, while thymosin *α*1 at approximately $100/1.6 mg, 3.2 mg/day for 7 days will increase the bill by $1400. Therefore, UTI for septic shock would add a considerable amount to patients' bill if they could not be covered by insurance. Consequently, the high cost might limit the popularity of UTI's application in septic shock. Nevertheless, if UTI could shorten ICU stay and mechanical ventilation time, as the data revealed in this meta-analysis, the extra cost of UTI might be well worth it.

The limitations of this systematic review and its meta-analyses are that all the RCTs were carried out in Asian countries such as Japan, China, South Korea, and India; the subjects were all Asian. Additionally, it is not clear whether the randomization process was adequately concealed/blinded in several RCTs. The lack of investigator blinding is a potential major flaw and source of potential bias that needs to be emphasized. Furthermore, with regard to the meta-analyses' results, only 28-day survival showed low heterogeneity, whereas APACHE II, ICU stay, and mechanical ventilation all showed considerable heterogeneity and random-model was used in the analytic process. That the average illness severity and age differed from trial to trial may be the source of heterogeneity; therefore, more well-designed trials in the near future are a feasible solution to remedy this flaw. Ulinastatin and thymosin *α*1 were widely used in the ICU field in Asia, but their efficacy was unclear up until now. While we do not intend to conclude that these two drugs are helpful, we are committed to drawing attention to them. Actually, we are somehow skeptical of the UTI's efficacy, since so far no guidelines exist recommending their application for sepsis. Regionally, UTI were mainly used in Asian countries, which made races of recipients relatively singular in the included trials. The average age of septic patients was usually above 50 years old or more, and drugs misused were either unhelpful or sometimes even harmful.

In sum, some big international multicenter RCTs are urgently needed to further prove UTI's efficacy. However, by the results of this meta-analysis, we might be able to conclude that with the treatment of UTI, the short-term survival rate might be able to be increased, illness severity might be ameliorated, and ICU stay and mechanical ventilation time might be reduced considerably in patients with sepsis—all of which might help hospitals lower medical costs and save limited medical resources.

## Figures and Tables

**Table 1:** Main characteristics of the studies included in the meta-analysis.

Reference	Design	Patients	Group	Population, ITT, *n* (treatment group versus control group)
			Dose, treatment duration	
Zhang et al. 2008 [14]	RCT (randomized and placebo controlled), patient blinded	Adult patients (18 years < age < 80 years) with confirmed sepsis	Ulinastatin, 200,000 U 3 times/day for 3 days; a subcutaneous dose of T*α*1, 1.6 mg, 2 times/day for 3 days followed by 100,000 U ulinastatin 3 times/day plus 1.6 mg T*α*1 once/day for 4 successive days. Placebo in the same way.	114 (59 versus 55)

Yumin et al. 2009 [15]	RCT (double-blinded placebo controlled clinical trial)	Adult patients with confirmed sepsis	For the first 3 days, 200K U ulinastatin and twice daily subcutaneous doses of 1.6 mg thymosin *α*1. For the next 4 days, intravenous doses of 100K U ulinastatin and twice daily subcutaneous doses of 1.6 mg thymosin *α*1. Placebo in the same way.	56 (23 versus 33)

Chen et al. 2009 [16]	Randomly assigned, placebo controlled, patient blinded	Adult patients (18 years < age < 80 years) with confirmed sepsis	200K U UTI 3 times per day plus a subcutaneous dose of 1.6 mg T*α*1 twice a day for 3 days followed by a dose of 100K U UTI thrice a day plus 1.6 mg T*α*1 once a day for four continuous days. Placebo in the same way.	114 (59 versus 55)

Huang et al. 2009 [17]	Randomized and placebo controlled, patient blinded	Adult patients (18 years < age < 80 years) with confirmed sepsis	200K U UTI 3 times per day plus a subcutaneous dose of 1.6 mg T*α*1 twice a day for 3 days followed by a dose of 100K U UTI thrice a day plus 1.6 mg T*α*1 once a day for four continuous days. Placebo in the same way.	70 (36 versus 34)

Lin 2007 [18]	Randomized and blank controlled, patient blinded	Adult patients (18 years < age < 80 years) with confirmed sepsis	600K U UTI intravenous injection once a day plus a subcutaneous dose of 3.2 mg T*α*1 once a day for 7 days. Blank control.	322 (164 versus 158)

Su et al. 2009 [19]	Randomized and blank controlled, patient blinded	Adult patients (18 years < age < 80 years) with confirmed sepsis	200K U UTI 2 times per day plus a subcutaneous dose of 1.6 mg T*α*1 twice a day for 4 days followed by a dose of 100K U UTI twice a day plus 1.6 mg T*α*1 once a day for 6 continuous days. Blank control.	242 (128 versus 114)
